# Ecdysteroid-containing food supplements from *Cyanotis arachnoidea* on the European market: evidence for spinach product counterfeiting

**DOI:** 10.1038/srep37322

**Published:** 2016-12-08

**Authors:** Attila Hunyadi, Ibolya Herke, Katalin Lengyel, Mária Báthori, Zoltán Kele, András Simon, Gábor Tóth, Kálmán Szendrei

**Affiliations:** 1 Institute of Pharmacognosy, University of Szeged, Eötvös str. 6, H-6720 Szeged, Hungary; 2 Department of Medical Chemistry, University of Szeged, Dóm Sq. 8, H-6720 Szeged, Hungary; 3 NMR Group, Department of Inorganic and Analytical Chemistry, Budapest University of Technology and Economics, Szt. Gellért Sq. 4, H-1111 Budapest, Hungary

## Abstract

Phytoecdysteroids like 20-hydroxyecdysone (“ecdysterone”) can exert a mild, non-hormonal anabolic/adaptogenic activity in mammals, and as such, are frequently used in food supplements. Spinach is well-known for its relatively low ecdysteroid content. *Cyanotis arachnoidea*, a plant native in China, is among the richest sources of phytoecdysteroids, and extracts of this plant are marketed in tons per year amounts via the internet at highly competitive prices. Here we report the investigation of a series of food supplements produced in Germany and claimed to contain spinach extracts. Twelve ecdysteroids including two new compounds were isolated and utilized as marker compounds. A comparative analysis of the products with Cyanotis and spinach extracts provides evidence that they were manufactured from Cyanotis extracts instead of spinach as stated. Based on the chromatographic fingerprints, 20-hydroxyecdysone 2- and 3-acetate are suggested as diagnostic markers for related quality control. This case appears to represent an unusual type of dietary supplement counterfeiting: undeclared extracts from alternative plants would supposedly ‘guarantee’ product efficacy.

Ecdysteroids, analogues of the insect moulting hormone ecdysone, have long been recognized for their multiple potential health benefits in mammals including humans. Accompanied by a negligible acute toxicity, these compounds can interact with a wide variety of metabolic processes, assist in maintaining the glucose and lipid homeostasis, and exert an overall “all-body strengthening” adaptogenic activity^1^,^2^. Due to their non-hormonal anabolic effect^3^ and the fact that they are not regulated as doping agents, these compounds have also drawn attention as possible performance enhancers in sports.

It is important to mention, that ecdysteroid composition of plants is generally dominated by a few major compounds (among which 20-hydroxyecdysone, 20E, is the far most typical) and a large variety of minor derivatives^4^. There are frequently orders of magnitude differences in the amounts of certain minor compounds and that of 20E within a plant. For example, large scale isolation of ecdysteroids from 1000 kg of *Leuzea carthamoides* yielded 1 kg of pure 20E, while further chromatographic purification of a selected crude fraction (still containing ca. 30% of 20E) gave minor compounds in 4–125 mg amounts^5^. Accordingly, it is not surprising that 20E is by far the most deeply studied ecdysteroid, and much less is known about the (supposedly, but not necessarily similar) bioactivities and safety of its less widespread derivatives.

Spinach, a worldwide popular and well-known vegetable has traditionally been considered as a food that would give strength: everyone has heard of Popeye and the source of his power. Since 1982, when our research group discovered the presence of ecdysteroids in spinach^6^, this traditional use can rationally be explained with the bioactivity of these compounds. On the other hand, spinach is a relatively poor ecdysteroid source: it contains ca. 0.005–0.08% of ecdysteroids (primarily 20E) per fresh weight depending on the time of harvest^7^, which means that even a “heavy-consumer” of spinach could hardly take more than a hundred milligrams of 20E per day from this source. On the other hand, sportsmen (most typically body-builders) are usually advised to take up to several grams of 20E per day, which dose has never seriously been studied for long-term safety.

In contrast with spinach, *Cyanotis arachnoidea*, a plant native in China, is undoubtedly among the richest sources of 20E; depending on the time and area of harvest, the levels of 20E in the roots can reach as high as 4–5%^8^. A simple internet search reveals dozens of companies offering several tons per year (some even claiming to be able to supply over a ton per week) which is available for internet-based purchase with a worldwide delivery. The scale of this market can well be demonstrated by the fact that some of these companies have a minimum order limit of 200 kg of “20E” or rather *C. arachnoidea* extract, and, depending on the purity, the price can be as low as around 10–20 USD per kg. Based on information obtained from the manufacturers, these extracts are offered for various uses: health benefits (including some questionable ones, *e.g.* antitumor properties) of ecdysteroids are typically advertised, and the use of such extracts for increasing the biomass production in agriculture and aquaculture is apparently also widespread.

Concerning any dietary uses, *C. arachnoidea* and its preparations have not been used in traditional herbal medicines or foodstuffs in Europe and it is unclear whether they can be legally marketed for human consumption^9^. In Hungary, high ecdysteroid containing plants, including a related species, *Cyanotis vaga* (together with *Achyranthes aspera, Cyathula capitata, Pfaffia paniculata* and *Polypodium virginianum*), are banned by the National Institute for Food and Nutrition Science (OÉTI) and, as such, cannot be marketed as food supplements^10^. It is also important to mention, that *C. arachnoidea* is neither a foodstuff nor a valued medicinal plant in China. Even though there are two ecdysteroids containing plant species in the Chinese Pharmacopoeia, such as *Cyathula officinalis* (Chuan Niuxi) and *Achyranthes bidentata* (Huai Niuxi), no Cyanotis species are recorded (Prof. De-an Guo; personal communication, 04.09.2015).

Due to decades-long experience of our group with phytoecdysteroids including the discovery of ecdysteroids’ presence in spinach^6^, the appearance of spinach-containing products marketed for their ecdysteroid content on the European market attracted our attention. Accordingly, our aim was to study the ecdysteroid composition of some of these products and to investigate the possibility of adulteration.

## Results and Discussion

### Analysis of ecdysteroid-containing food supplements

**Figure 1:**
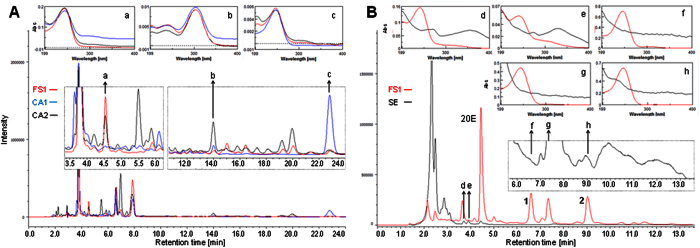
(**A**) HPLC fingerprints of FS1 (red), the mother liquid of CA1 (blue) and CA2 (black). a, b and c: UV spectra at Rt = 4.53, 14.08 and 22.88 min, respectively; chromatographic conditions: Kinetex Biphenyl 4.6 × 250 mm, 5 μm, 25% ACN (aq), flow: 1 mL/min. (**B**) HPLC fingerprints of FS1 (red) and the spinach extract prepared by us (SE; black); d, e, f, g and h: UV spectra at Rt = 3.72, 3.92, 6.59, 7.33 and 9.03 min, respectively; chromatographic conditions: Kinetex XB-C18 4.6 × 250 mm, 5 μm, 52% MeOH (aq), flow: 1 mL/min. **1**: 20E 2-acetate, **2**: 20E 3-acetate. The qualitative fingerprints of FS1, CA1 and CA2 are nearly perfect match, while in FS1 and SE no common major constituents can be detected.

A quick analysis of the purchased products by thin-layer chromatography (TLC) immediately revealed such a complex-pattern ecdysteroid content, which seemed extremely unlikely for spinach. Following this, the HPLC fingerprints of the food supplements were compared with those of two independent samples of *C. arachnoidea* extracts purchased from China (CA1 and CA2), as well as with that of an authentic spinach extract prepared by us. Selected chromatograms for the product FS1 are presented in Fig. 1.

It can well be seen from Fig. 1A, that not only the main accompanying ecdysteroids but also numerous minor constituents of *C. arachnoidea* are perfect match to those of FS1, only the relative amounts are different. Additionally, no ecdysteroids of FS1, other than 20E, were detectable within the spinach extract, nor could any characteristic constituents of spinach be observed within FS1 except for 20E itself (Fig. 1B). One could certainly argue that FS1 might still be originated from spinach, and the differences we observe come from a different extraction procedure or different processing techniques, *e.g.* solid-phase extraction (SPE) or column chromatography that could have been applied in order to enrich its originally low ecdysteroid content. There are simple techniques to separate ecdysteroids from phenolic constituents, *e.g.* on polyamide the two flavonoid-like compounds observed close to the peak of 20E within spinach could likely be removed^11^, and the large amount of highly polar constituents of spinach could also be removed by SPE on (quite expensive) reversed-phase silica. No such processing would, however, explain why not even trace amounts of any of the minor or accompanying major ecdysteroids of FS1 are detectable in an authentic sample of spinach. This, together with the matching overall chromatographic fingerprint of FS1 to those of two independent samples of Cyanotis extracts, provides evidence that the food supplements were manufactured using *Cyanotis arachnoidea* instead of spinach.

### Isolation and structure elucidation of ecdysteroids from food supplement FS1

**Figure 2:**
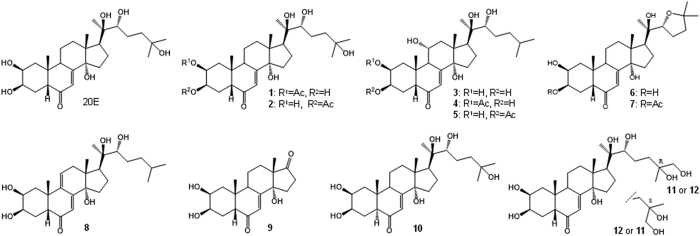
Ecdysteroids isolated from product FS1. 20E: 20-hydroxyecdysone, **1**: 20-hydroxyecdysone 2-acetate, **2**: 20-hydroxyecdysone 3-acetate, **3**: ajugasterone C, **4**: ajugasterone C 2-acetate, **5**: ajugasterone C 3-acetate, **6**: shidasterone, **7**: shidasterone 3-acetate, **8**: dacryhainansterone, **9**: rubrosterone, **10**: 5α-20-hydroxyecdysone, **11** and **12**: 20,26-dihydroxyecdysone – although **11** and **12** were isolated in pure form, their configuration at C-25 could not be assigned by NMR, **4** and **7** are new compounds.

Following the above observations, our next aim was to isolate the major and possibly the minor ecdysteroids of *C. arachnoidea* by utilizing FS1, the product which was proven to be a rich raw material for such an initiative. By processing 5 g of FS1 with the consecutive use of various chromatographic methods of different selectivity, twelve ecdysteroids other than 20E were successfully isolated including two new compounds (**4** and **7**). Chemical structures of the two new isolates were elucidated by comprehensive one- and two-dimensional NMR methods allowing a complete signal assignment. ^1^H and ^13^C NMR data of the new ecdysteroids **4** and **7** are presented in Table 1, along with those of shidasterone (**6**), the non-acetylated reference compound for **7**. The new NMR data obtained in methanol-*d*_*4*_ for compounds **11** and **12** are compiled in Table 2. Chemical structures of the isolated compounds are presented in Fig. 2.

The ^1^H and ^13^C NMR spectra of compounds **1–3**, **5**, **6**, and **8–12** confirmed that these compounds are identical with 20E 2-acetate (**1**), 20E 3-acetate (**2**), ajugasterone C (**3**), ajugasterone C 3-acetate (**5**), shidasterone (**6**), dacryhainansterone (**8**), rubrosterone (**9**), 5α-20-hydroxyecdysone (**10**), and 25*R*- and 25-*S*-20,26-dihydroxyecdysone (**11** and **12**).

In case of compound **4**, twenty-nine ^13^C signals were discernible in the DEPTQ spectrum, indicating the presence of six methyl, seven methylene, and nine methyne groups, in addition to one quaternary sp^2^ (δ 165.7) and four quaternary sp^3^ carbon atoms, and one conjugated C=O (δ 206.0) and one O-C=O (δ 172.6) by moiety. Integration of the signals in the ^1^H NMR spectrum obtained in methanol-*d*_4_ summed up to 41 H atoms. Considering the molecular formula of C_29_H_46_O_8_ established by means of HRMS, the molecule contains five –OH substituents. The number of double bond equivalents in compound **4** is seven; therefore this compound contains four rings and three double bonds. Utilizing edited HSQC and sets of different selective TOCSY and ROESY experiments, we achieved a complete ^1^H and ^13^C signal assignment of **4** as well as its stereo structure was revealed. All ^1^H and ^13^C chemical shifts are nearly identical with those of ajugasterone C^12^ with the exception of the H-C(2) signals, namely that the original δH-2: 4.01 and δC-2: 68.9 ppm values changed into δH-2: 5.13 and δC-2: 73.4, verifying the presence of an AcO-2 group (δCH_3_ 2.05 s, (δ O=C-CH_3_ 172.6 and 21.3) instead of a HO-2. NMR spectra of compound **4** are available as Supplementary Information.

In case of compound **7**, the molecular formula of C_29_H_46_O_7_ was established by HRMS. The NMR analysis revealed twenty-nine ^13^C signals, six methyl, eight methylene, seven methyne groups, furthermore five quaternary sp^3^ and one quaternary sp^2^ carbon (δ 168.4) atoms, and one conjugated C=O (δ 205.6) and one O-C=O (δ 172.6) by moiety. Integration of the signals in the ^1^H NMR spectrum summed up to 41H atoms. Considering the molecular formula, the molecule contains three –OH groups. Thus, compound **7** contains five rings and three double bonds. As also demonstrated in Table 1, the ^1^H and ^13^C chemical shifts of this steroid skeleton were nearly identical with those of shidasterone^12^ with the exception of the H-C(3) signals, namely that the original δH-3: 3.95 and δC-3: 68.7 values changed into δH-3: 5.15 and δC-3: 71.9. This verified the presence of an AcO-3 group (δCH_3_ 2.11 s, (δ O=C-CH_3_ 172.6 and 21.2) instead of a HO-3. Due to the small quantity of compound **7**, the HMBC spectrum was utilized for identification of the chemical shifts of quaternary carbon atoms. NMR spectra of compound **7** are available as Supplementary Information.

The isolation and synthesis of the two C-25 stereoisomers of 20,26-dihydroxyecdysone (**11** and **12**) were previously reported^13^; these two compounds were distinguished from each-other by their ^1^H and ^13^C NMR data taken in pyridine-*d*_5_. In the present work, our comprehensive one- and two-dimensional NMR study performed in methanol-*d*_*4*_ resulted a complete ^13^C NMR and ^1^H signal assignment also including the differentiation of the H_α_ and H_β_ methylene signals. A comparison of the ^1^H and ^13^C chemical shifts of the C-25 epimers revealed that they were almost identical. The only characteristic difference in the spectra was observed at the signals of the CH_2_-OH group, namely in compound **11** the δH_2_-27 signal appeared as a singlet at 3.37 (2H) and δC-27 appeared at 70.8 ppm, whereas in case of **12** the δH_2_-27 signal appeared in form of dublets at 3.39 (1H) and 3.35 (1H), and δC-27 appeared at 70.2 ppm. It is noteworthy that the NMR results do not permit the evaluation of the absolute configuration of both epimers. Representative NMR spectra of compounds **11** and **12** are available as Supplementary Information.

Compounds **4** and **7** are new ecdysteroids. However, no sound judgment concerning their natural origin or artefact nature can be made at this point considering that the source was an ecdysteroid containing product not directly the plant itself. The presence of several ecdysteroid acetates, mainly those of 2-acetyl substituted ones, might be of chemotaxonomical interest: according to the regularly updated online ecdysteroid database^12^, only five ecdysteroid 2-acetates have been isolated from plants till now, and three of them from Cyanotis species. Ajugasterone C 2-acetate (**4**) might be the fourth in this series, assuming that its natural (i.e. not artefact) origin will be proven. Anyhow, its presence in the marketed spinach products, together with that of 20-hydroxyecdysone 2-acetate (**1**), is further evidence supporting that these products originate from a Cyanotis plant. Considering these phytochemical aspects and that compounds **1** and **2** appear as major peaks in the chromatographic fingerprints of both CA1 and CA2 but not in that of spinach (see Fig. 1B), 20E 2-acetate (**1**) and 20E 3-acetate (**2**) are hereby suggested as chemical marker compounds for a diagnostic identification of Cyanotis extracts within ecdysteroid-containing products.

### Quantitative analysis of food supplements FS1-5

The amounts of the major isolated ecdysteroids within products FS1-5 were determined by RP-HPLC-DAD, results of this study are compiled in Table 3.

Based on our results, the following approximate doses are ingested with a recommended daily intake of 2 capsules. 20E: 2–24 mg; **1**: ≤9 mg; **2**: ≤11.7 mg; **4**: 0.2–0.8 mg; **6**: 0.1–0.9 mg; **8**: 0.3–1.8 mg; **10**: ≤0.3 mg; **11 **+ **12**: ≤0.7 mg. These doses are in fact well within the limit generally considered safe for 20E *per os*. On the other hand, it must be emphasized that neither European traditional knowledge nor safety studies are available for Cyanotis species. The roots of *Cyanotis arachnoidea* are not considered dietary anywhere, and practically nothing is known on the other constituents of the plant or their safety, in contrast with the case of spinach. We believe that mentioning the tragic story that has become well-known as the “Chinese herb nephropathy”, exerted by the aristolochic acid content of certain supposedly safe herbal remedies^14^, is sufficient to highlight the potential danger in marketing the extracts of uninvestigated plant species for human consumption on the sole basis of the known safety of one single constituent group (ecdysteroids).

## Conclusions

Our results provide evidence that the investigated food supplements contain Cyanotis extract instead of spinach as claimed. This case appears to represent an unusual type of dietary supplement counterfeiting: the alleged pharmacological activities are supposedly enhanced by the addition of extracts from undeclared plants that contain higher amounts of the same active ingredient(s). Although this may be to “guarantee” product efficacy, such an act may bypass European dietary supplement regulations (correct declaration of composition, dosage, safety, etc.).

It needs to be highlighted that the current study investigated only one single product family from a single provider. Large quantities of ecdysteroid-enriched Cyanotis extracts are available on the market at inexpensive prices, but at this point it is difficult to make a sound judgment on how much this type of counterfeiting is spread within the EU. Since neither *C. arachnoidea* nor other Cyanotis species have a history of human consumption in Europe, any products containing such extracts may need to be subject to proper authorization. At the same time, our results highlight the urgency of in-depth safety studies on Cyanotis extracts.

## Methods

### Plant material and sample preparation

Two independent preparations of *C. arachnoidea* have been purchased: CA1 (90% claimed purity of 20E by means of HPLC, Shaanxi KingSci Biotechnology Co., Ltd., Shanghai, China) and CA2 (50% claimed purity of 20E by means of UV absorbance, Xi’an Olin Biological Technology Co., Ltd., Xi’an, China). CA1 was re-crystallized from ethyl acetate–methanol (2:1, v/v). The mother liquid of this process, enriched in minor constituents, was utilized together with CA2 for comparison with the food supplements. Fresh spinach leaves were purchased from the local food market in Szeged, Hungary in July, 2014. Extraction was performed by sonication in methanol, and the re-dissolved crude extract was utilized for analysis after evaporation under reduced pressure at 40 °C.

### Ecdysteroid containing food supplements

A series of ecdysteroid-containing products claimed to contain spinach extract were purchased by ordering from the producer, VerdeVital Beratungs-, Import- und Vertriebsgesselschaft mbH (Bovenden, Germany) in 2013. These products were VerdeFit (L13761012), VerdeKlimEx (L09760711), VerdeDry (L09810711), VerdeOs (L09800711) and VerdeArthroSan (L00250113), and hereby they are referred to as FS1-5, respectively. Herbal constituents of FS1-5 per capsules were claimed as follows. FS1: spinach extract (SE) (440 mg); FS2: SE (400 mg), isoflavones from Soy (20 mg); FS3: SE (300 mg), Cranberry extract (200 mg); FS4: SE (250 mg); FS5: SE (250 mg).

### Thin-layer chromatography (TLC)

Ecdysteroid composition of samples was studied by analytical TLC on Silica gel 60 F_254_ plates (Merck, Darmstadt, Germany), by utilizing solvent systems of Toluene – acetone – ethanol – cc ammonia (100:140:32:9, v/v/v/v) or ethyl acetate – ethanol – water (16:2:1, v/v/v). Ecdysteroids were detected as absorbing spots under 254 nm UV light, and, after spraying the plates by vanillin sulphuric acid reagent (0.5 g of vanillin in 100 mL of 98% sulfuric acid), spots of various colours under visible light and fluorescent spots under 366 nm UV light.

### Chemicals

Chromatographic solvents of analytical or HPLC grade were obtained from Molar Chemicals, Budapest, Hungary, Sigma-Aldrich Kft, Budapest, Hungary, or Avantor Performance Materials, Gliwice, Poland. Ultrapure water was obtained from a Millipore Direct-Q 3 UV equipment (Millipore S.A.S, Molsheim, France). Vanillin was obtained from Reanal Private Ltd., Budapest, Hungary.

### Isolation of ecdysteroids from FS1

After removing from the capsules, 20 g powder of FS1 was extracted with 3 × 100 mL of MeOH by sonication for 10 min and the solvent was evaporated under reduced pressure at 40 °C. The dry residue of the extract (3.1 g) was fractionated by flash chromatography on an equipment of two BÜCHI C605 pumps controlled by a C615 pump manager (BÜCHI Labortechnik AG, Flawil, Switzerland) with a stepwise gradient of 30–60% of aqueous MeOH on a 40 × 150 mm C_18_ column at a flow rate of 60 mL/min, collecting 70 fractions of 50–75 mL. The fractions were joined based on their TLC fingerprints, and these joint fractions were further purified by a multi-step procedure of rotational planar chromatography (RPC) on a Chromatotron equipment (Harrison Research, Palo Alto, CA, USA) by using stepwise gradients from MeOH – CH_2_Cl_2_ (25:1, v/v) to MeOH – CH_2_Cl_2_ – benzene (25:3:2, v/v/v) or from EtOAc – EtOH (40:1, v/v) to EtOAc – EtOH – H_2_O (40:2.5:1, v/v/v) on 1 or 2 mm thin silica layers. In order to maximize yields, parallel RPC sub-fractions originating from neighboring fractions of the first flash chromatographic separation were compared and joined whenever high similarities in the TLC fingerprints were observed. After one or two consecutive RPC purification steps, compounds were isolated by NP- or RP-HPLC on a Jasco equipment containing two PU2080, an MD-2010 Plus diode-array detector and an AS2055plus autosampler (Jasco Ltd., Tokyo, Japan). HPLC conditions were as follows: Zorbax-Sil 250 × 9.6 mm, 5 μm column, flow: 3 mL/min with a solvent system of hexane – i-propanol – H_2_O (100:50:4, v/v/v) for compounds **1**, **2**, **4**, **5**, **7** and **8** (NP-HPLC) or Gemini-Nx C_18_ 250 × 10 mm, 5 μm column, flow 3 mL/min with a solvent system of 65% aqueous MeOH (compound **6**), 40% aqueous MeOH (compound **10**) or 13% aqueous acetonitrile (ACN) for separating compounds **11** and **12** (RP-HPLC). Compound **3** was obtained from RPC in pure form and **9** was crystallized from an RPC fraction. Isolated amounts of the compounds were as follows: **1** (8.8 mg), **2** (25.5 mg), **3** (4.5 mg), **4** (13.6 mg), **5** (5.0 mg), **6** (4.9 mg), **7** (0.8 mg), **8** (5.4 mg), **9** (23.1 mg), **10** (3.3 mg), **11** (1.2 mg), **12** (1.8 mg).

### NMR spectroscopy

^1^H (500.1 MHz) and ^13^C (125.6 MHz) NMR spectra were recorded at room temperature on a Bruker 500 Avance III spectrometer equipped with a cryo probehead, or a Bruker Avance 500 (Bruker Biospin Co., Karlsruhe, Germany) spectrometer. Amounts of approximately 0.8–5 mg of the compounds were dissolved in 0.1 mL of methanol-*d*_4_ and transferred to 2.5 mm Bruker MATCH NMR sample tube. Chemical shifts are given on the δ-scale and are referenced to the solvent (methanol- *d*_4_: δC = 49.1 and δH = 3.31 ppm). Pulse programs of all experiments (^1^H, ^13^C, DEPTQ, DEPT-135, sel-TOCSY (mixing time: 120 ms), sel-ROE (300 ms), gradient-selected (gs) ^1^H,^1^H-COSY, edited gs-HSQC, gs-HMBC (optimized for 10 Hz), ROESY (350 ms) were taken from the Bruker software library. For 1D measurement, 64 K data points were used to yield the FID. For 2D measurements, sweep width in F2 was 4000 Hz; all data points (t2 × t1) were acquired with 2 K × 256. For F1, linear prediction was applied to enhance the resolution. Most ^1^H assignments were accomplished using general knowledge of chemical shift dispersion with the aid of the proton-proton coupling pattern (^1^H NMR spectra). The NMR signals of the products were assigned by comprehensive one- and two-dimensional NMR methods using widely accepted strategies^15^,^16^.

### High-resolution mass spectrometry (HRMS)

HRMS spectra were recorded on a Q Exactive Plus hybrid quadrupole-orbitrap mass spectrometer (Thermo Scientific, Waltham, MA, USA) equipped with a heated electrospray ionization (HESI-II) probe used in positive mode and coupled to an Acquity UPLC (Waters, Saint-Quentin, France).

### Analytical HPLC and quantitative determination of ecdysteroids in FS1-5

Products FS1-5, Cyanotis samples CA1 and CA2, and spinach extract were compared by analytical scale RP-HPLC-DAD on the above-mentioned Jasco HPLC system by using two columns of different selectivity, a Zorbax Eclipse XDB-C8, 150 × 4.6 mm, 5 μm (Agilent Technologies, Santa Clara, CA, USA), and a Kinetex XB-C18, 250 × 4.6 mm, 5 μm (Phenomenex, Torrance, CA, USA), both with aqueous MeOH and aqueous ACN as solvents. For quantifying compounds **1**, **2**, **4**, **6**, **7** and **10**–**12** within the five products, a single point experiment was performed on the above-mentioned Kinetex column, with a gradient system of 17% aqueous ACN increasing to 30% in 15 min, then to 55% in 2 min which composition stayed for 2 min and subsequently returned to 17% of ACN. The flow rate was 1 mL/min. Six-point calibration was performed by injecting 10 μL of standard solutions containing 500, 125, 31.25, 7.81, 1.95 or 0.49 μg/mL of 20E, prepared by four-times serial dilutions from a 2 mg/mL stock solution hence the results are expressed in equivalents of 20E, and integration was performed automatically at λ = 245 nm at a slope sensitivity of 100.00 μV/sec.

## Additional Information

**How to cite this article**: Hunyadi, A. *et al*. Ecdysteroid containing food supplements from *Cyanotis arachnoidea* on the European market: evidence for spinach product counterfeiting. *Sci. Rep.* **6**, 37322; doi: 10.1038/srep37322 (2016).

**Publisher's note:** Springer Nature remains neutral with regard to jurisdictional claims in published maps and institutional affiliations.

## Supplementary Material

Supplementary Information

## Figures and Tables

**Table 1:** ^1^H and ^13^C chemical shifts, multiplicities and coupling constants of compounds 4, 6 and 7 in methanol-*d*_4._

Atom no.	4^a^	*J* (Hz)	C	6	*J* (Hz)	C	7^b^	*J* (Hz)	C
	H			H			H		
1βα	1.622.56	t; 12.5dd; 12.5, 4.0	36.1	1.431.79	t; 12.5	37.5	1.421.93	t; 12.5	38.6
2	5.13	ddd; 12.5, 4.0, 3.0	73.4	3.84	dt; 12.5, 3.5	68.8	3.97	dt; 12.5, 3.5	67.2
3	4.18	q; ~3.0	65.8	3.95	q; ~3.0	68.7	5.15	q; ~3.5	71.9
4βα	1.681.84	ddd; 13.0, 4.0, 3.0td; 13.0, 3.0	33.3	1.731.73		33.0	1.771.77		30.4
5	2.39	dd; 13.0, 4.0	52.6	2.38	dd; 12.8, 4.8	51.9	2.22	dd; 12.5, 5.0	52.7
6	—		206.0	—		206.5	—		205.6
7	5.82	d; 2.5	122.8	5.81	d; 2.7	122.2	5.82	d; 2.5	122.1
8	—		165.7	—		168.1	—		168.4
9	3.19	dd; 9.0, 2.5	43.0	3.15		35.2	3.16	ddd, 12.0, 6.5, 2.5	35.3
10	—		40.0	—		39.4	—		39.8
11βα	4.09—	ddd; 11.5, 9.0, 6.0	69.6	1.681.81		21.6	1.691.82		21.7
12βα	2.152.22	dd; 12.0, 6.0t; ~12.0	43.7	1.852.15	td; 13.0, 5.0	32.4	1.872.16	td; 13.0, 5.0	32.4
13	—		48.7	—		48.4	—		48.5
14	—		85.0	—		85.4	—		85.3
15βα	1.971.58		31.9	1.951.60		31.8	1.971.60		31.9
16βα	1.981.73		21.6	1.991.70		21.9	2.001.82		21.8
17	2.42	t; 9.0	50.4	2.37	t; 9.0	52.0	2.37	t; 9.0	51.9
18	0.88	s	19.0	0.85	s	18.2	0.85	s	18.2
19	1.09	s	24.7	0.96	s	24.5	0.99	s	24.4
20	—		77.9	—		77.1	—		77.2
21	1.19	s	21.1	1.21	s	20.9	1.22	s	20.8
22	3.32		78.0	3.92	dd; 8.5, 6.2	85.7	3.92	dd; 8.5, 6.0	85.6
23	1.571.23		30.6	1.891.76		28.5	1.901.75		28.6
24	1.471.23		37.8	1.751.75		39.7	1.751.75		39.7
25	1.57		29.3	—		81.9	—		81.9
26	0.92	d; 6.5	22.9	1.24	s	28.4	1.24	s	28.5
27	0.93	d; 6.5	23.6	1.25	s	29.1	1.25	s	29.1

^a^AcO-2 ^1^H: 2.05 s; ^13^C 21.3, 172.6.

^b^AcO-3 ^1^H: 2.11 s; ^13^C 21.2, 172.6.

**Table 2:** ^1^H and ^13^C chemical shifts, multiplicities and coupling constants of compounds 11 and 12 in methanol-*d*_4_.

Atom no.	11	*J* (Hz)	C	12	*J* (Hz)	C
	H			H		
1βα	1.441.80	t; 12.5	37.2	1.431.80	t; 12.5	37.2
2	3.84	ddd; 12.5, 4.5, 3.5	68.8	3.84	ddd; 12.5, 4.5, 3.5	68.8
3	3.95	q; ~3.5	68.6	3.95	q; ~3.5	68.6
4βα	1.761.70		33.0	1.761.71		33.0
5	2.38	dd; 12.5, 4.5	51.9	2.38	dd; 12.5, 4.5	51.9
6	—		206.3	—		206.3
7	5.81	d; 2.5	122.2	5.81	d; 2.5	122.2
8	—		168.0	—		168.0
9	3.15	ddd; 11.0, 7.0, 2.5	35.2	3.15	ddd; 11.0, 7.0, 2.5	35.2
10	—		39.3	—		39.3
11βα	1.701.81		21.6	1.731.81		21.6
12βα	1.882.14	td; 13.0, 5.0	32.6	1.882.13	td; 13.0, 5.0	32.6
13	—		48.6	—		48.6
14	—		85.3	—		85.3
15βα	1.971.60		31.9	1.971.59		31.9
16βα	2.001.74		21.6	1.981.73		21.6
17	2.39	t; 8.0	50.6	2.38	t; 8.5	50.6
18	0.89	s	18.1	0.89	s	18.1
19	0.97	s	24.5	0.97	s	24.5
20	—		77.7	—		77.7
21	1.20	s	21.1	1.20	s	21.1
22	3.34	dd; 10.7, 1.7	77.7	3.33	dd; 10.7, 1.7	77.7
23	1.691.28		26.6	1.651.29		26.6
24	1.791.46		37.2	1.821.45		37.2
25	—		73.7	—		73.7
26	1.14	s	23.6	1.15	s	23.6
27	3.37	s	70.8	3.393.35	d; 11.0d; 11.0	70.2

**Table 3:** Amounts of the individual compounds in the investigated products by means of calibration with 20E (R^2^ = 0.9999).

20E equivalents (m/m%)									
Product	20E	1	2	4	6	8	10	11 or 12	12 or 11
FS1	2.40	0.90	1.17	0.08	0.09	0.18	0.03	0.02	0.02
FS2	0.28	0.27	0.26	0.04	0.03	0.08	*	*	*
FS3	0.29	0.21	0.30	0.02	0.01	0.04	*	0.07	*
FS4	0.20	*	*	0.02	0.01	0.03	0.01	0.02	*
FS5	1.11	0.30	0.41	0.03	0.04	0.07	0.02	*	*
