# Supplementary material for: Ecdysteroid-containing food supplements from Cyanotis arachnoidea on the European market: evidence for spinach product counterfeiting
Source: Sci Rep. 2016 Dec 8;6:37322. doi: 10.1038/srep37322 (PMC5144001; doi:10.1038/srep37322)

Supplementary Information

for

Ecdysteroid containing food supplements from *Cyanotis arachnoidea* on the European market – how to fake spinach?

Attila Hunyadi [a,*], Ibolya Herke [a], Katalin Lengyel [a], Mária Báthori [a], Zoltán Kele [b], András Simon [c], Gábor Tóth [c], Kálmán Szendrei [a]

[a] Institute of Pharmacognosy, University of Szeged, Eötvös str. 6, H-6720 Szeged, Hungary
[b] Department of Medical Chemistry, University of Szeged, Dóm Sq. 8, H-6720 Szeged, Hungary
[c] NMR Group, Department of Inorganic and Analytical Chemistry, Budapest University of Technology and Economics, Szt. Gellért Sq. 4, H-1111 Budapest, Hungary

Table of Contents

NMR spectra – compound **4**……………………………………………………2 - 4

NMR spectra – compound **7**……………………………………………………5 - 7

NMR spectra – compound **11** and **12**……………………………………………..8 - 9

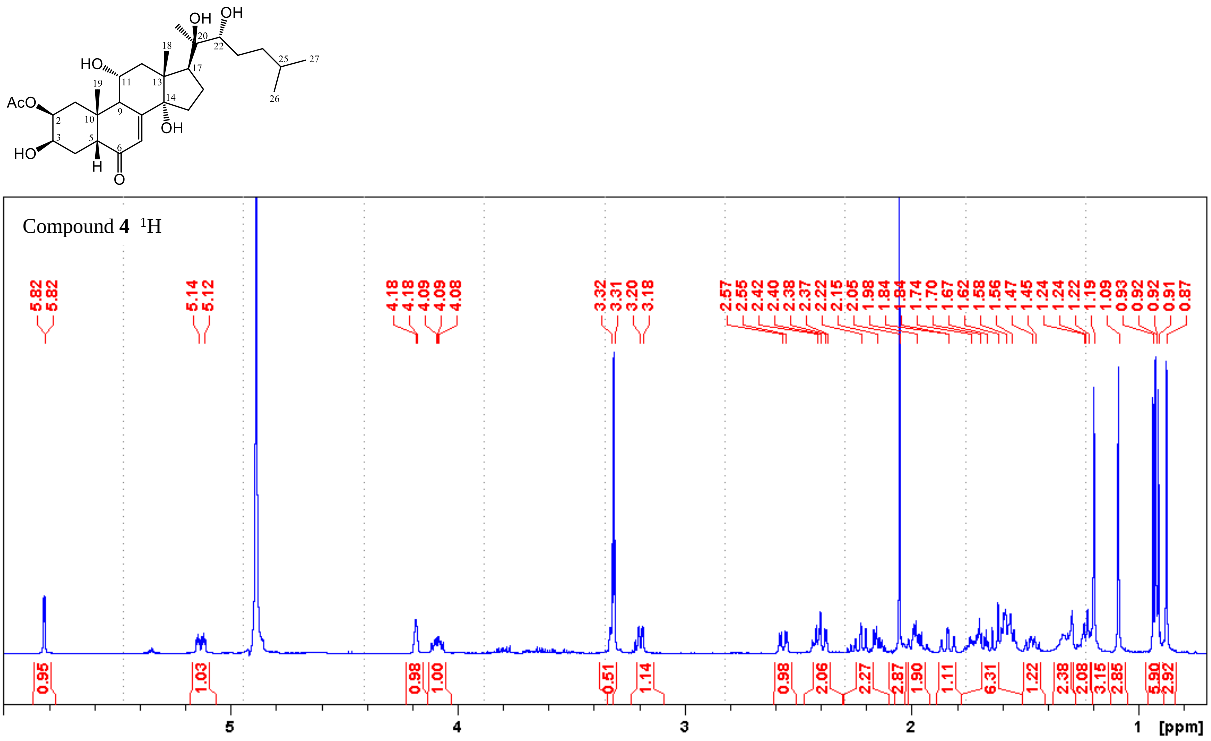
Compound 4 1H
[ppm]

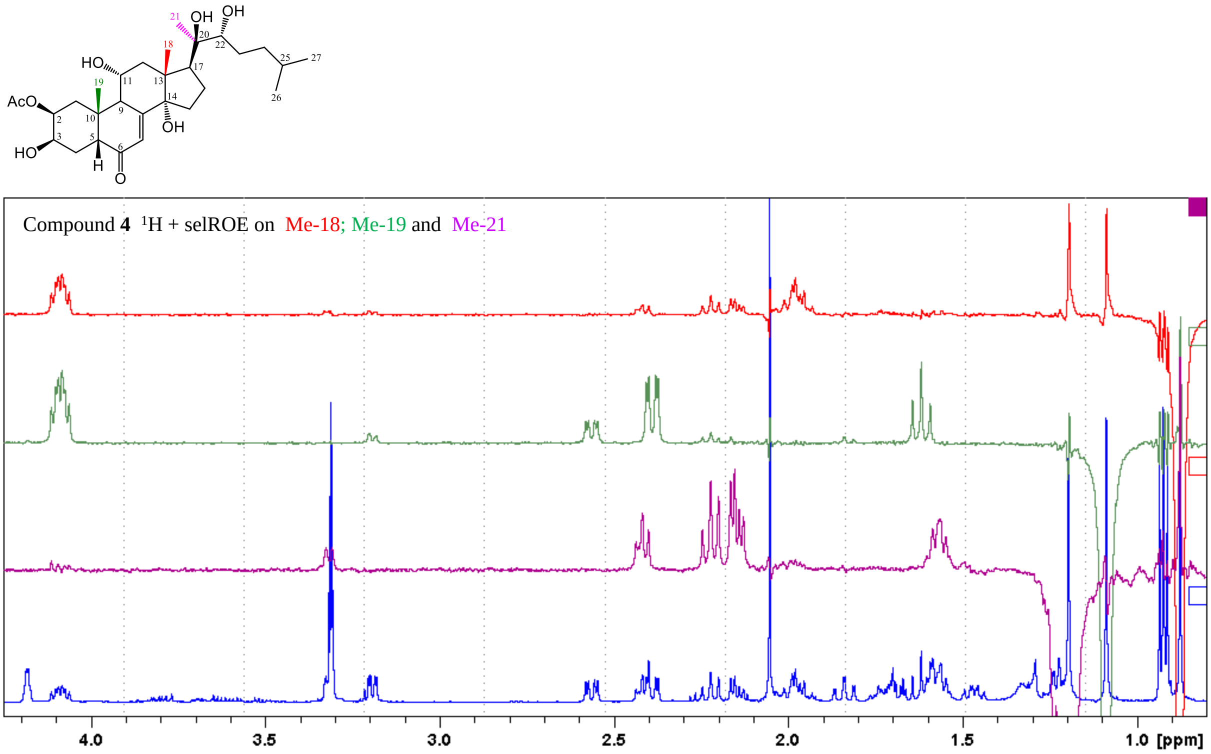
Compound 4 1H + selROE on Me-18; Me-19 and Me-21
[ppm]

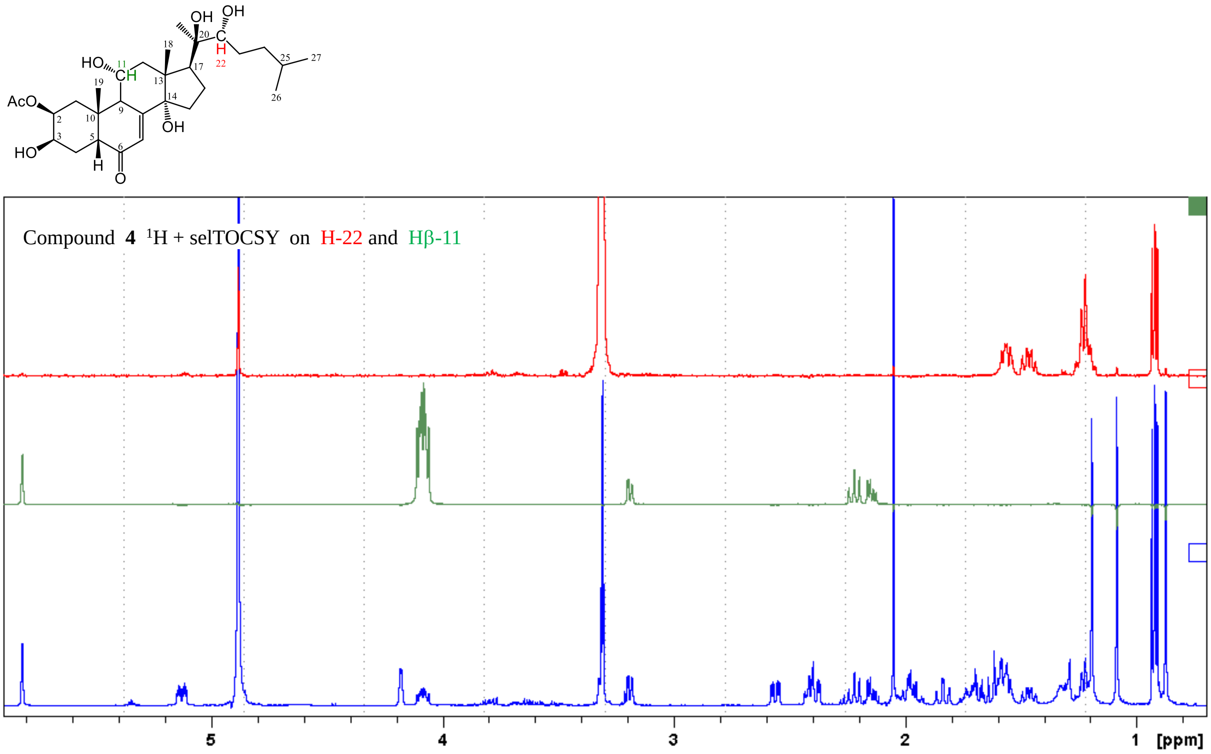
Compound 4 1H + selTOCSY on H-22 and Hβ-11
[ppm]

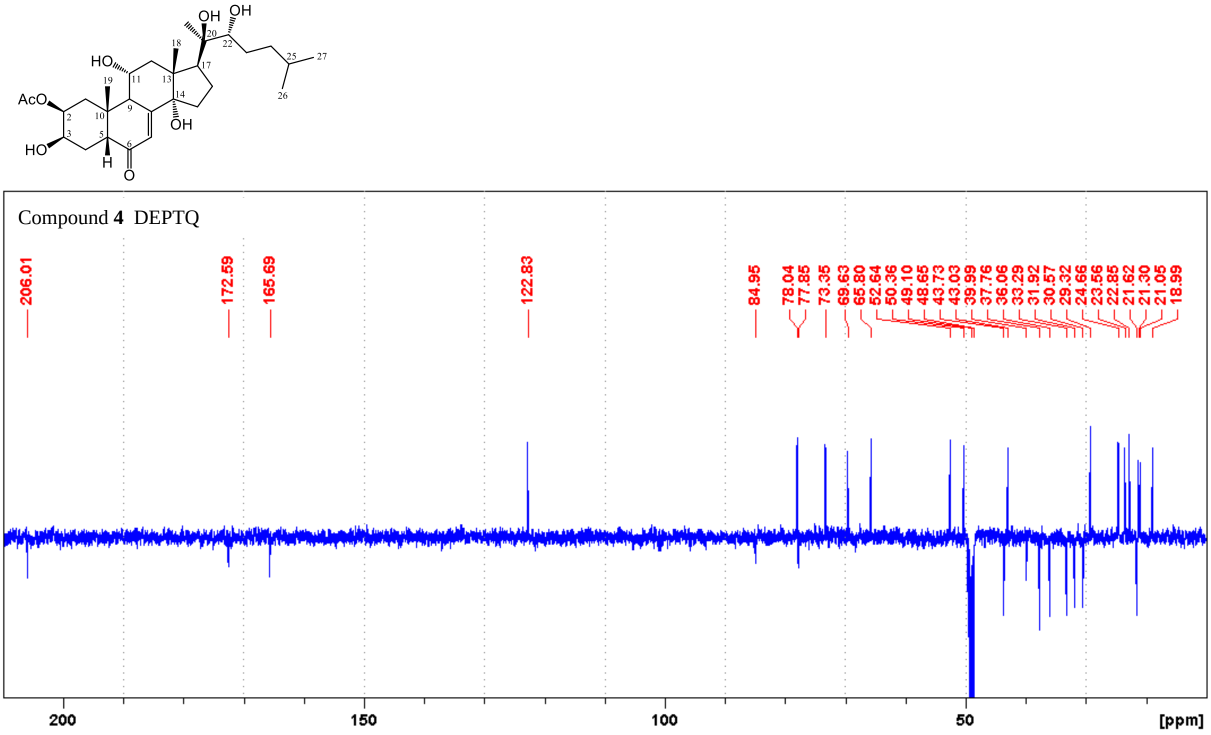
Compound 4 DEPTQ
206.01
172.59
165.69
122.83
84.95
78.04
77.85
73.35
69.63
65.80
52.64
50.36
49.10
48.65
43.73
43.03
39.99
37.76
36.06
33.29
31.92
30.57
29.32
24.66
23.56
22.85
21.62
21.30
21.05
18.99
[ppm]

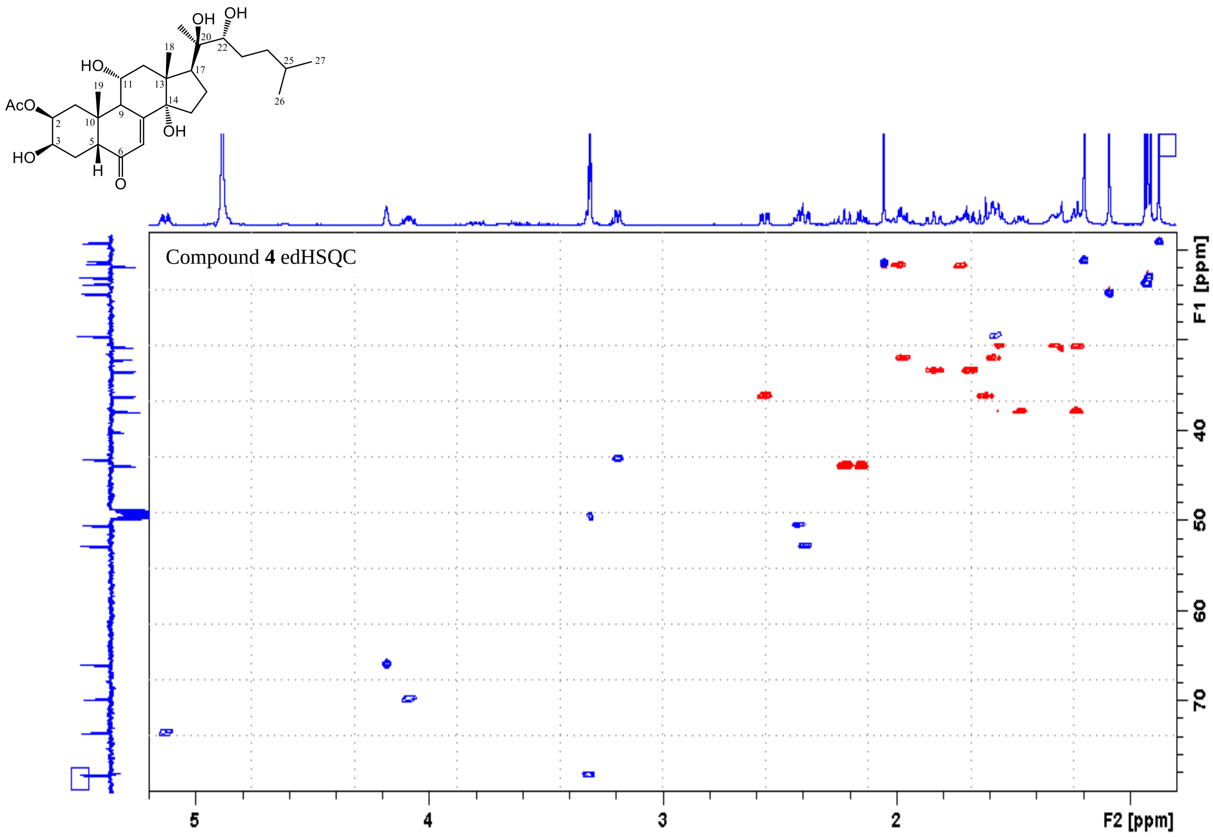
Compound 4 edHSQC
F1 [ppm]
F2 [ppm]

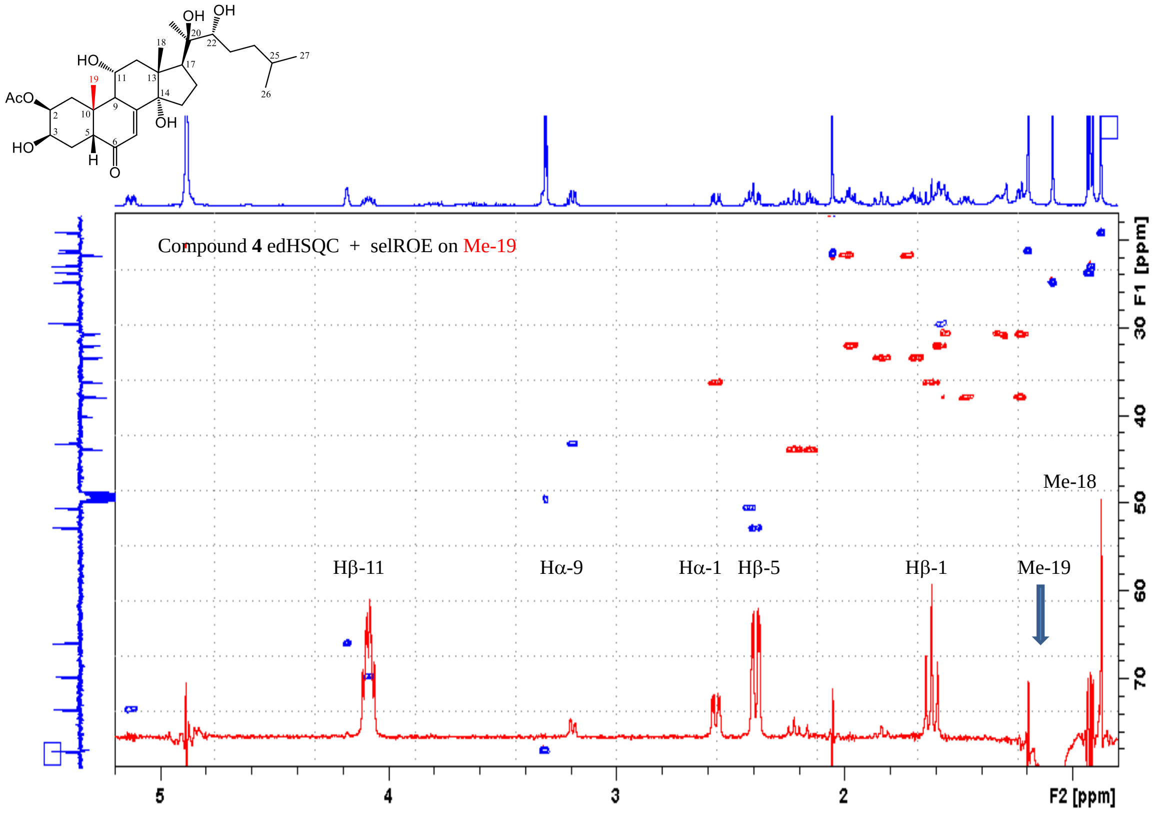
Compound 4 edHSQC + selROE on Me-19
Hβ-11
Hα-9
Hα-1
Hβ-5
Hβ-1
Me-19
Me-18
F1 [ppm]
F2 [ppm]

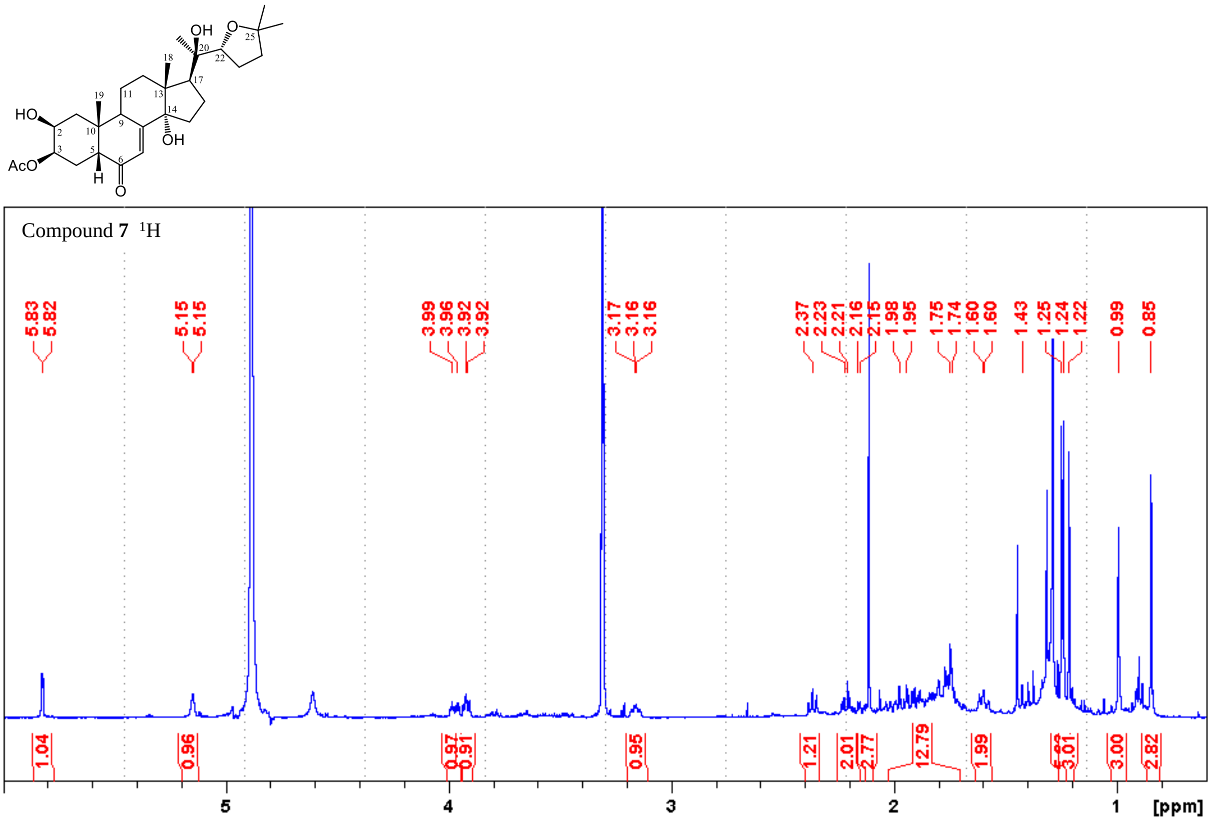

Compound **7** ^{1}H

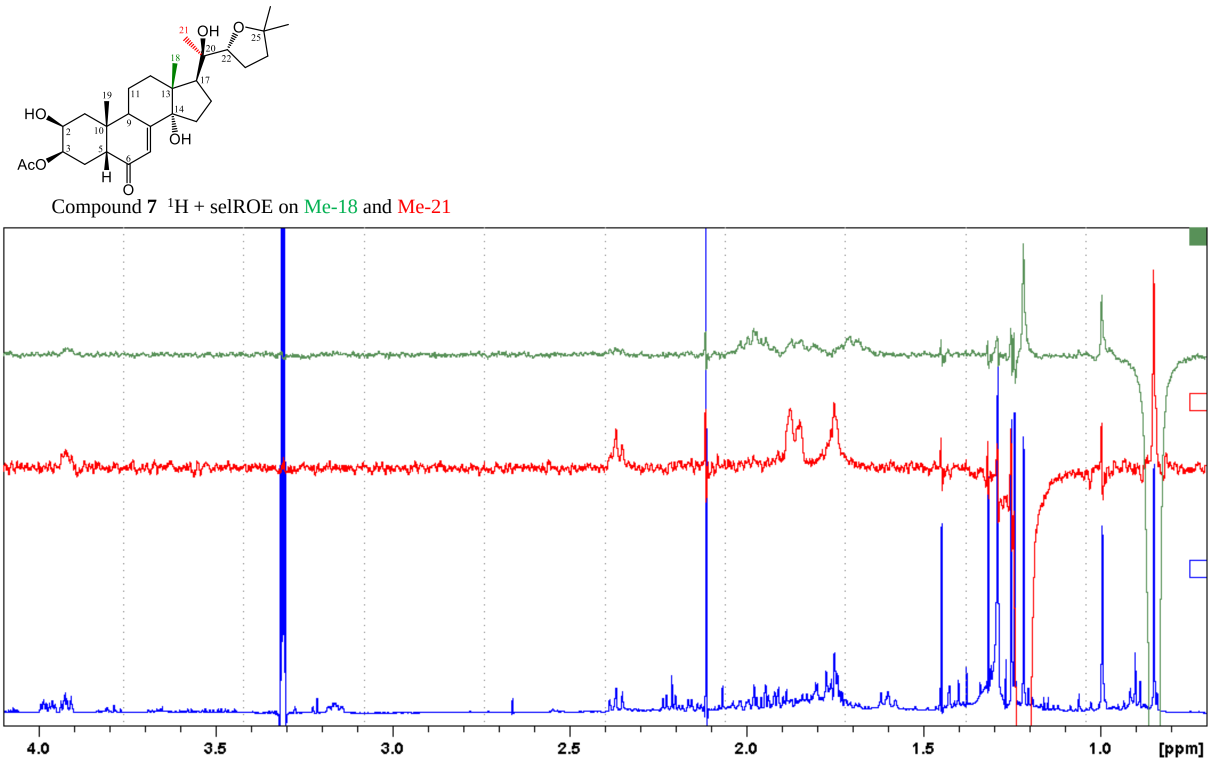

Compound **7** ^{1}H + selROE on Me-18 and Me-21

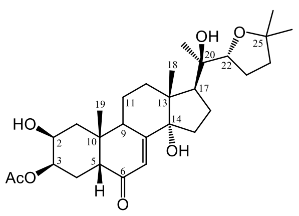
OH
O
25
20
22
18
17
19
11
13
HO
14
2
10
9
OH
AcO
3
5
6
H
O

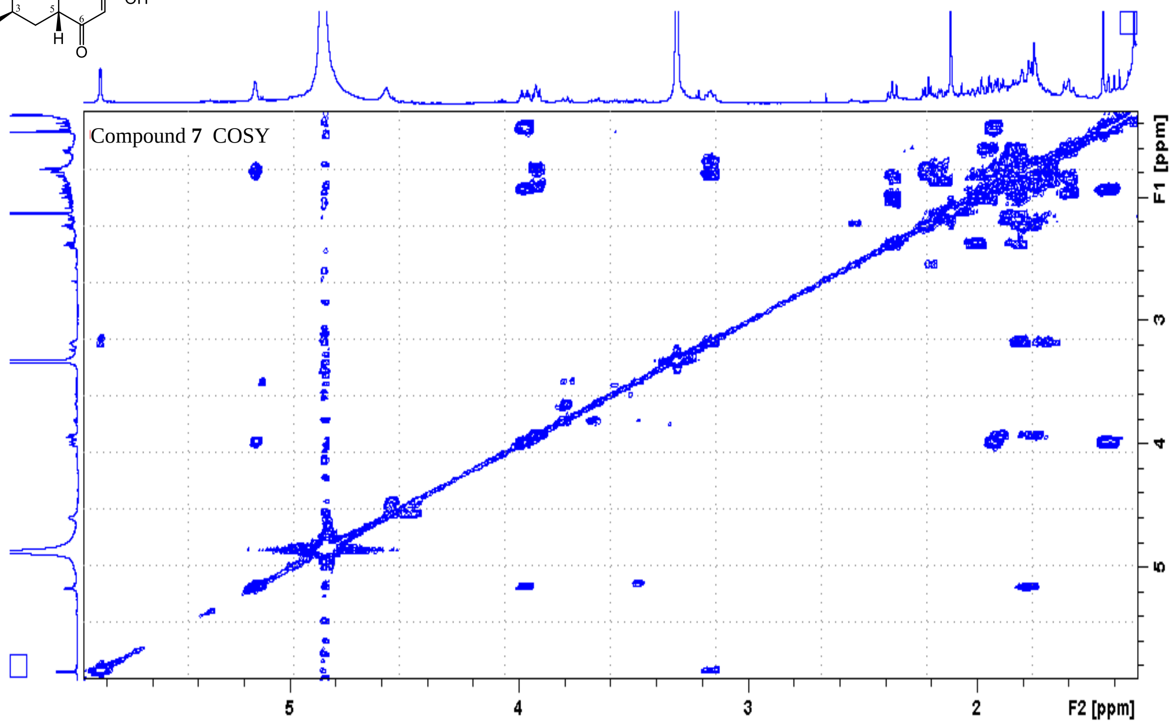
Compound 7 COSY
F1 [ppm]
3
4
5
5
4
3
2
F2 [ppm]

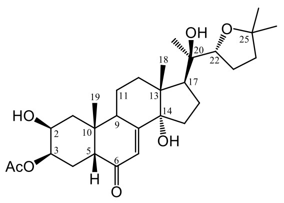
OH
O
25
20
22
18
17
19
11
13
HO
14
2
10
9
OH
AcO
3
5
6
H
O

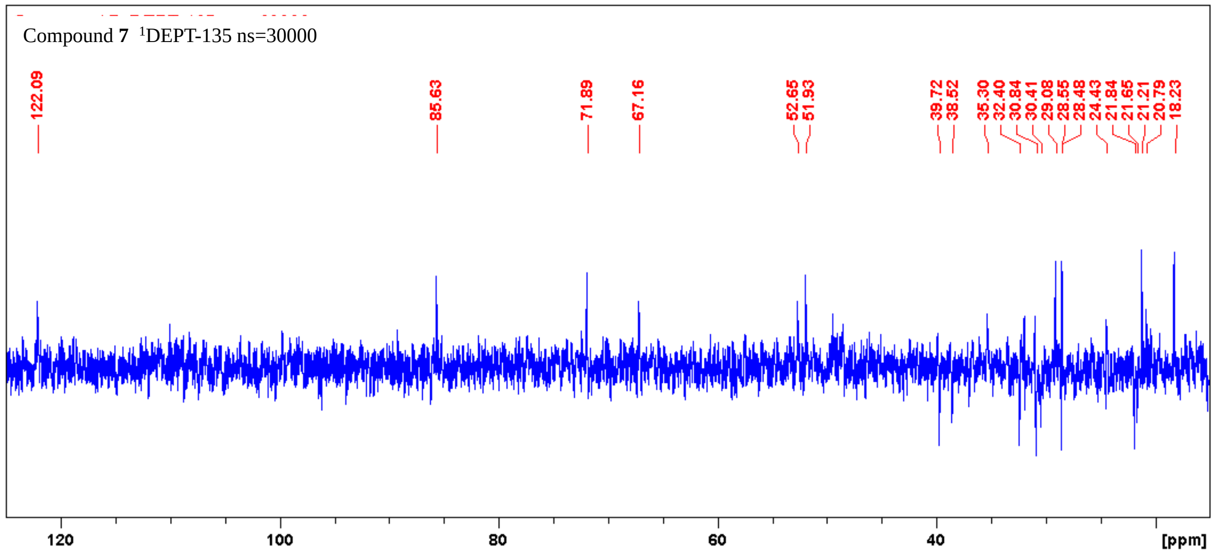
Compound 7 1DEPT-135 ns=30000
122.09
85.63
71.89
67.16
52.65
51.93
39.72
38.52
35.30
32.40
30.84
30.41
29.08
28.55
28.48
24.43
21.84
21.65
21.21
20.79
18.23
120
100
80
60
40
[ppm]

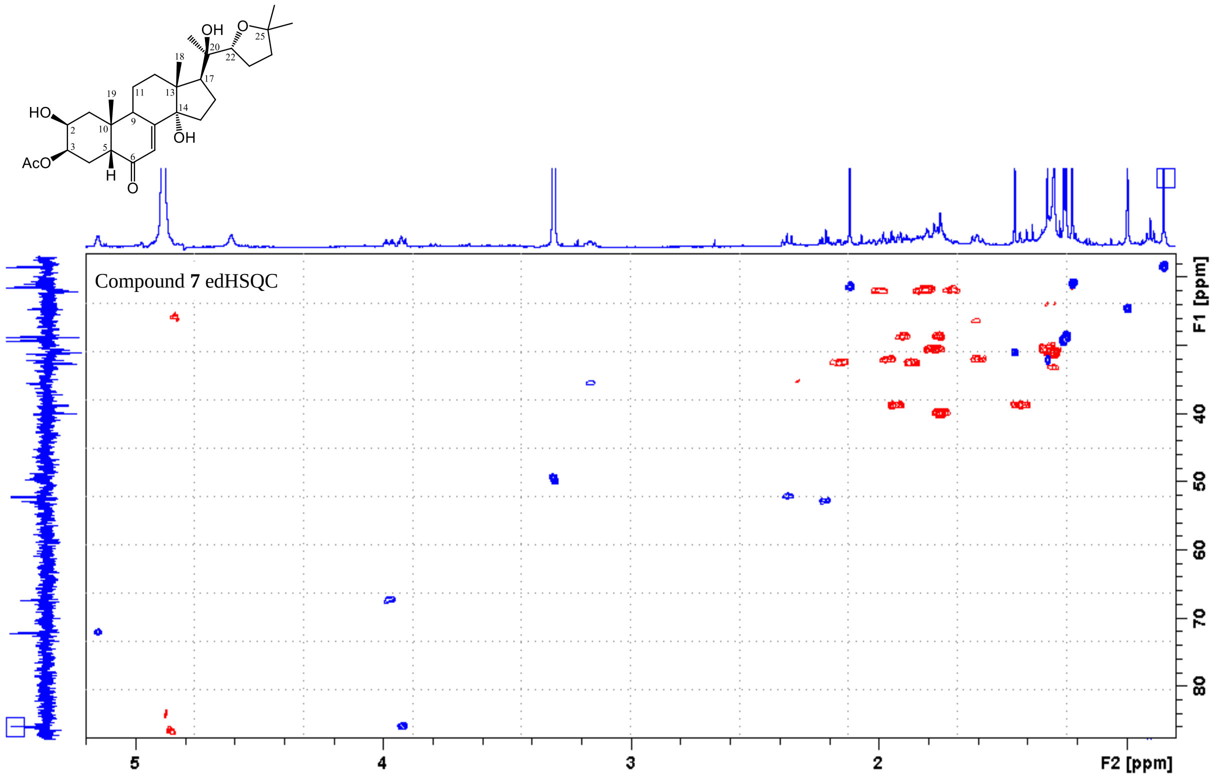
Compound 7 edHSQC
F1 [ppm]
F2 [ppm]
HO
AcO
OH
O
H

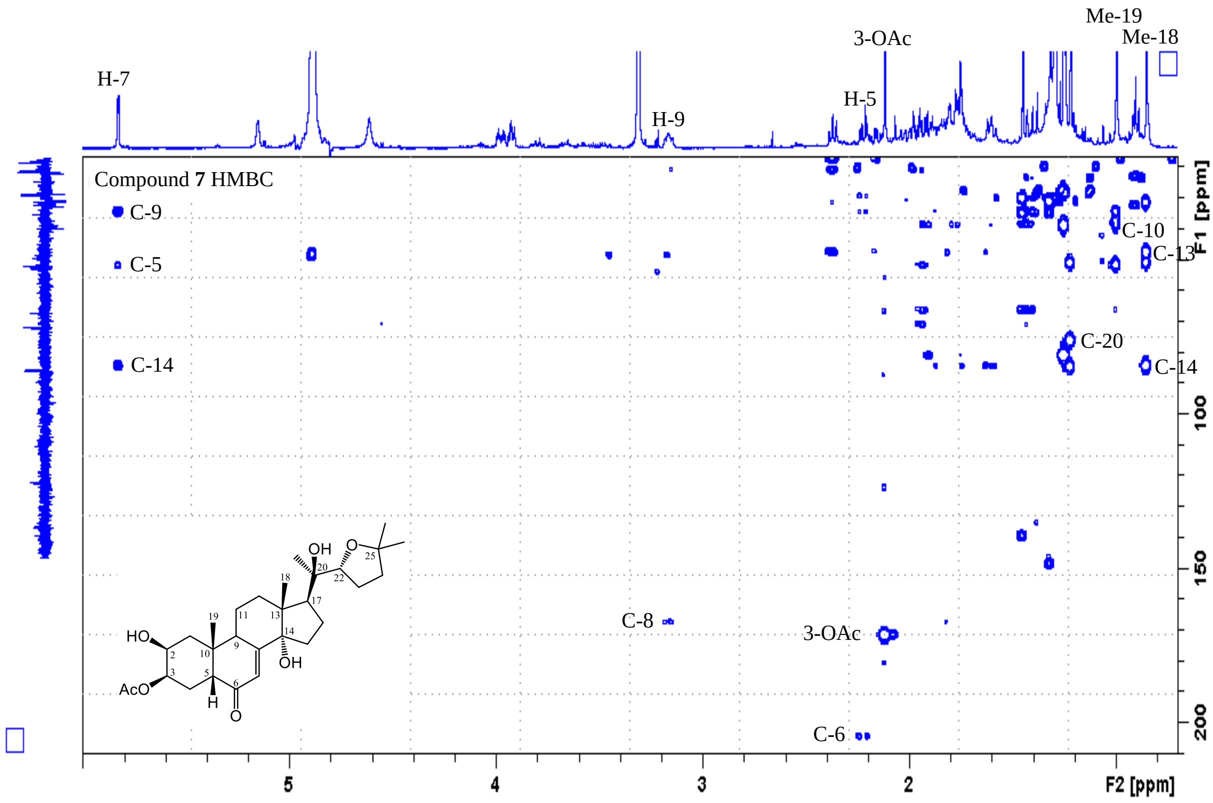
Compound 7 HMBC
H-7
H-9
H-5
3-OAc
Me-19
Me-18
C-9
C-5
C-14
C-10
C-13
C-20
C-14
C-8
3-OAc
C-6
F1 [ppm]
F2 [ppm]

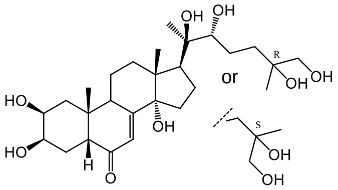
OH
OH
R
or
OH
OH
HO
HO
H
O
OH
S
OH
OH

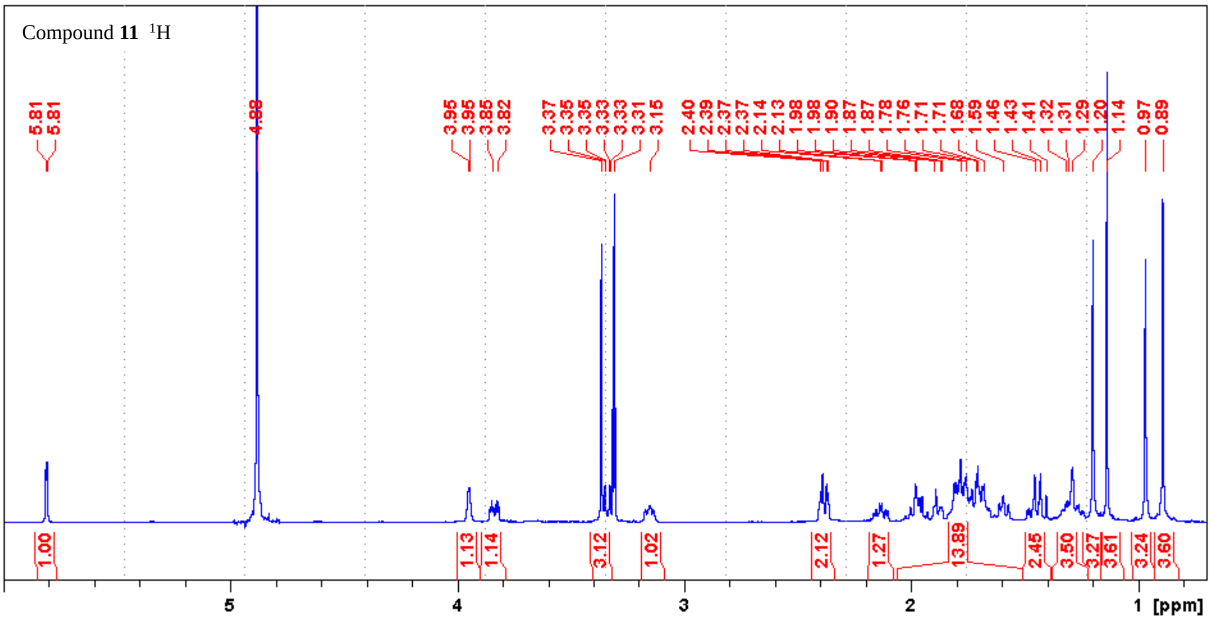
Compound 11 1H
5.81
5.81
4.88
3.95
3.95
3.85
3.82
3.37
3.35
3.35
3.33
3.33
3.31
3.15
2.40
2.39
2.37
2.37
2.14
2.13
1.98
1.90
1.87
1.87
1.78
1.76
1.71
1.71
1.68
1.59
1.46
1.43
1.41
1.32
1.31
1.29
1.20
1.14
0.97
0.89
1.00
1.13
1.14
3.12
1.02
2.12
1.27
13.89
2.45
3.50
3.27
3.61
3.24
3.60
5
4
3
2
1 [ppm]

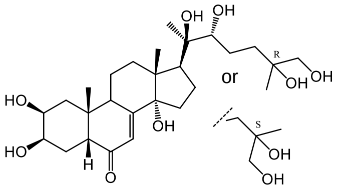
OH
OH
R
or
OH
OH
HO
HO
H
O
OH
S
OH
OH

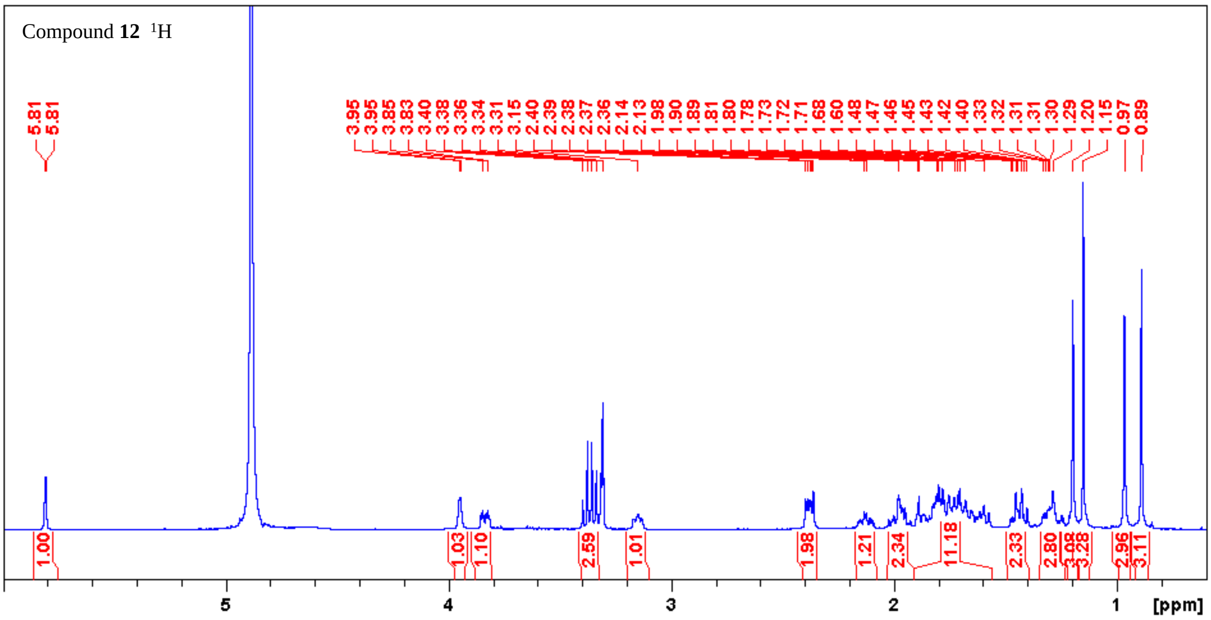
Compound 12 1H
5.81
5.81
3.95
3.95
3.85
3.83
3.40
3.38
3.36
3.34
3.31
3.15
2.40
2.39
2.38
2.37
2.36
2.14
2.13
1.98
1.90
1.89
1.81
1.80
1.78
1.73
1.72
1.71
1.68
1.60
1.48
1.47
1.46
1.45
1.43
1.42
1.40
1.32
1.31
1.30
1.29
1.20
1.15
0.97
0.89
1.00
1.03
1.10
2.59
1.01
1.98
1.21
2.34
11.18
2.33
2.80
3.08
3.28
2.96
3.11
5
4
3
2
1
[ppm]

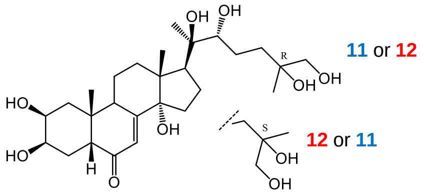

Compounds **11** and **12**; sections of ^{1}H spectra

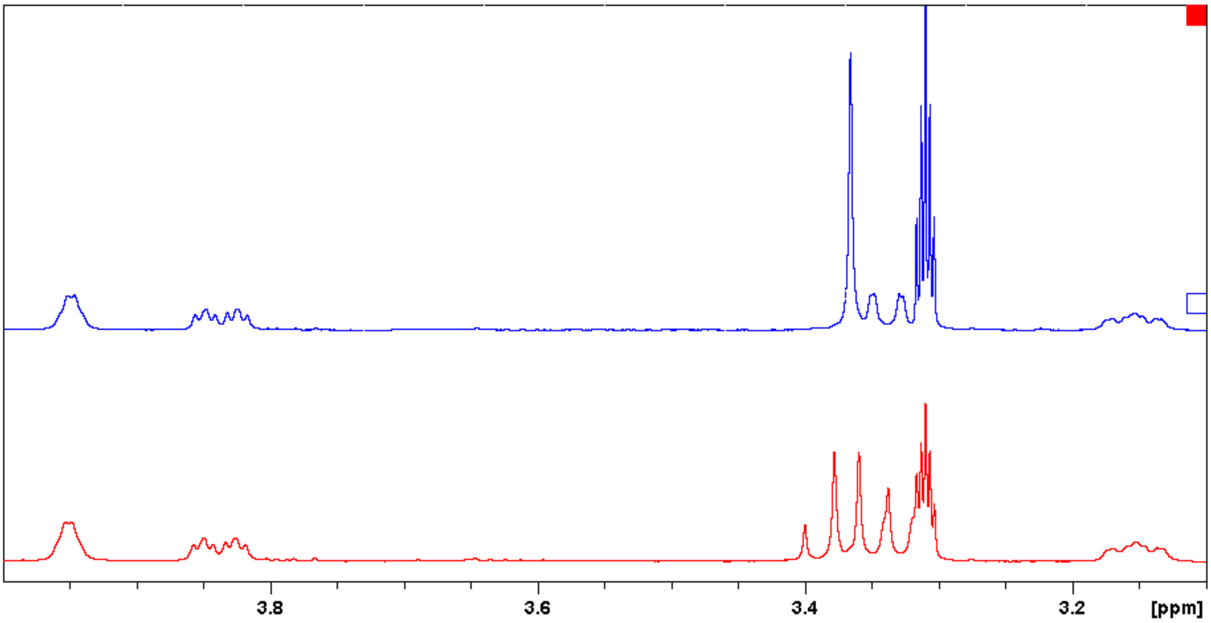

Supplement: Supplementary Information [file srep37322-s1.pdf]
